# Capture of Part of a Vessel Wall by a Coronary Orbital Atherectomy System

**DOI:** 10.1016/j.cjco.2022.09.004

**Published:** 2022-09-29

**Authors:** Hidetoshi Yasuda, Kikuo Isoda, Shinya Okazaki, Tohru Minamino

**Affiliations:** a Department of Cardiovascular Biology and Medicine, Juntendo University Graduate School of Medicine, Tokyo, Japan; b Department of Cardiology, Juntendo University Nerima Hospital, Tokyo, Japan

**Figure 1:**
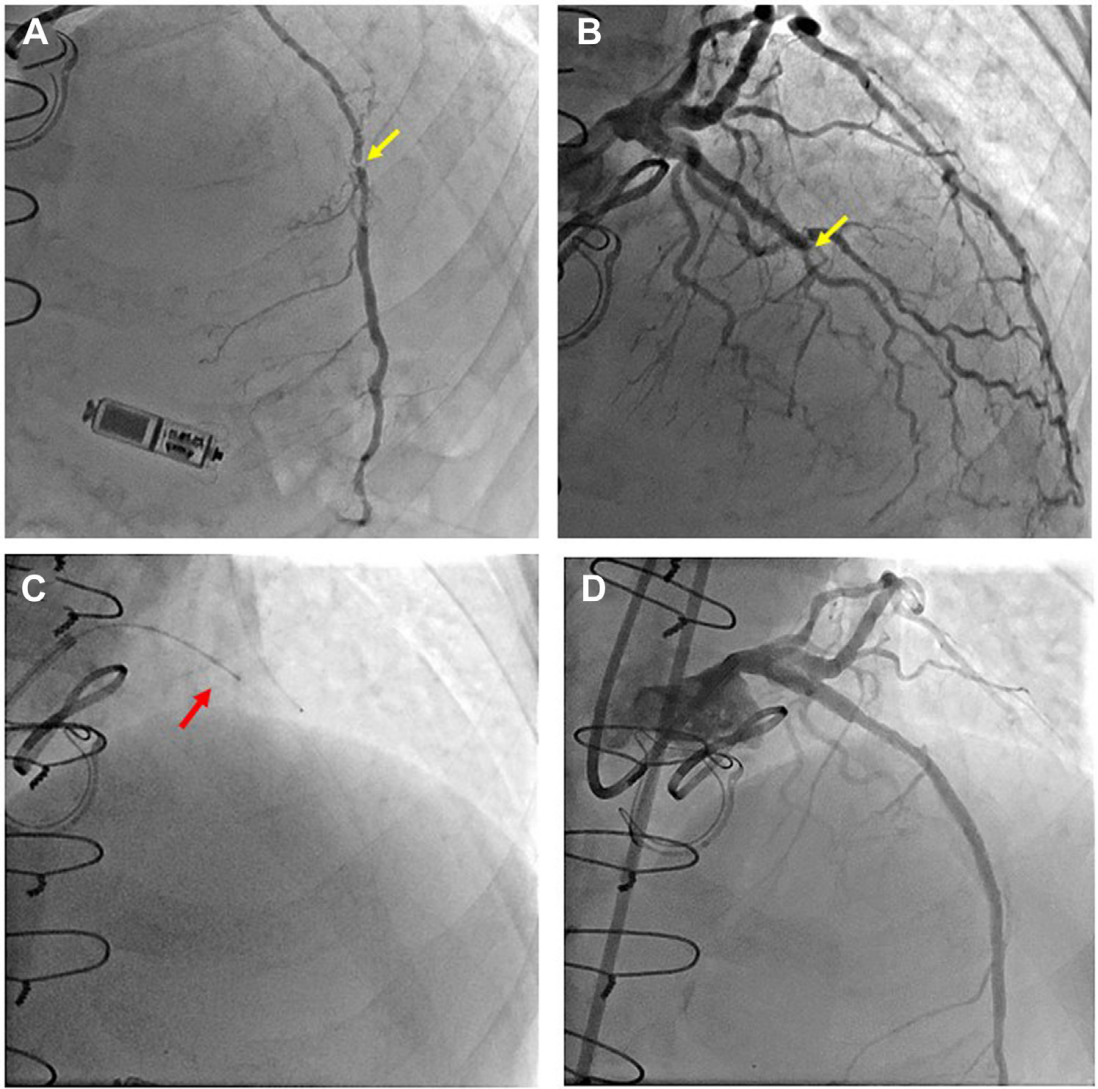
(**A**) Initial internal thoracic angiogram (**yellow arrow** indicates the stenotic lesion) and (**B**) coronary angiogram (**yellow arrow** indicates the total occlusion site). (**C**) **Red arrow** indicates the orbital atherectomy system tip. (**D**) Final angiogram.

**Figure 2:**
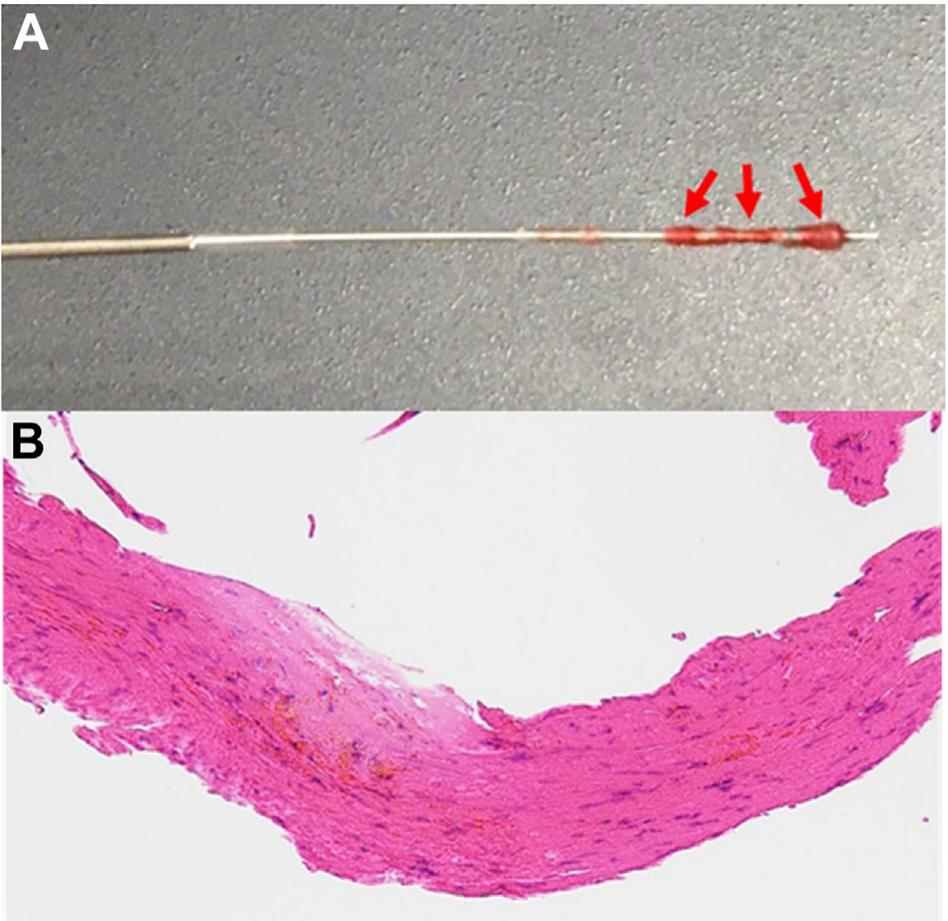
(**A**) Uptake of tissue by orbital atherectomy system tip (**red arrows**). (**B**) Image of histologic section of the tissue stained with hematoxylin and eosin (×40).

An 82-year-old man who had undergone coronary artery bypass grafting, aortic/mitral valve replacement, and tricuspid valvuloplasty was seen for exertional angina. On angiography, a severely stenotic lesion of the distal left internal thoracic artery (LITA) was noted, which was a bypass graft to the left anterior descending (LAD) artery (Fig. 1A, arrow). The LAD artery showed chronic total occlusion (CTO; Fig. 1B, arrow). We thought that if an angioplasty for the stenotic lesion in the LITA failed, then significant damage would occur in the LAD artery area. Therefore, the decision was made to perform a percutaneous coronary intervention for the CTO of the proximal LAD artery. We were able to cross a wire using contralateral injection from a microcathether in the LITA. After crossing a wire through the CTO lesion, an intravascular ultrasound (IVUS) catheter could not be passed through the lesion, and predilatation was performed with a 1.5- × 15-mm balloon. Then, a Diamondback 360 Coronary Orbital Atherectomy System (OAS, Medikit Co, Tokyo, Japan; Cardiovascular Systems; Fig. 1C, arrow) was used, because the lesion had severe calcification and the wire was seen on IVUS to have passed through the true lumen. Some resistance was felt while advancing the OAS, and the OAS could not be passed through the lesion. The OAS was removed from the guiding catheter, and a large dissection extending from the proximal LAD artery up to the left main coronary artery was evident on IVUS. No ischemia or hemodynamic compromise occurred, however, because the LAD artery flow was supplied from the bypass graft. Fortunately, we were able to perform successful bailout stenting for OAS-induced coronary dissection involving the left main coronary artery (Fig. 1D) after additional dilatation for the CTO lesion with a 2.5- × 15-mm noncompliant balloon. After the intervention, we noted that several bits of tissue were clinging to the crown and the drive shaft of the OAS (Fig. 2A, arrows), and pathologic examination confirmed that the tissue was part of the vessel wall (Fig. 2B). In addition, investigation of the causes of this issue revealed that strong friction was present between the wire and the OAS tip. This finding suggests that if resistance is encountered while pressure is applied to an OAS, the procedure should be stopped and the OAS checked, because variation of the ablation diameter depends largely on the resistance of the tip. An OAS uses a differential sanding mechanism of action designed to reduce plaque while minimizing damage to the vessel wall.^1^ However our report demonstrates the mechanism dose not always work. Although several cases of OAS tip rupture have been reported previously,^2^ this report is the first that includes images of an accidental OAS uptake of part of a vessel wall.

**Novel Teaching Points**

• The differential sanding mechanism of an OAS does not always work and can cause tissue rupture.
• The variation in ablation diameter depends largely on the resistance of the tip of an OAS.
• If resistance is encountered while an OAS is in use, the procedure should be stopped and the OAS should be checked.
